# Prolapse or incontinence: what affects sexual function the most?

**DOI:** 10.1007/s00192-015-2887-2

**Published:** 2015-11-19

**Authors:** Swati Jha, Deepa Gopinath

**Affiliations:** Department of Urogynaecology, Sheffield Teaching Hospitals NHS Foundation Trust, Jessop Wing, Tree Root Walk, Sheffield, UK S10 2SF; Stepping Hill Hospital, Stockport NHS Foundation Trust, Poplar Grove, Stockport, UK SK2 7JE

**Keywords:** Urinary incontinence, Pelvic organ prolapse, Sexual function, Sexual dysfunction, Pelvic floor dysfunction

## Abstract

**Introduction and hypothesis:**

Pelvic organ prolapse (POP) and stress urinary incontinence (SUI) adversely affect sexual function in women. Comparative studies of the two subgroups are few and results are conflicting. The aim of this study was to compare the effect of POP and SUI on the sexual function of women undergoing surgery for these conditions.

**Methods:**

The study population comprised women with POP or SUI in a tertiary referral hospital in the UK. Women who underwent SUI surgery had no symptoms of POP and had urodynamically proven stress incontinence. Patients with POP had ≥ stage 2 prolapse, without bothersome urinary symptoms. Pre-operative data on sexual function were collected and compared using an electronic pelvic floor assessment questionnaire (ePAQ). The incidence of sexual dysfunction and comparison of symptoms in both groups were calculated using the Mann–Whitney* U* test.

**Results:**

Three hundred and forty-three women undergoing surgery for either SUI or POP were included. Patients were age-matched, with 184 undergoing SUI surgery (age range 33–77 years) and 159 POP surgery (age range 27–78 years; *p* = 0.869). The overall impact of POP and SUI was not significantly different in the two subgroups (*p* = 0.703). However, both patients (73 % vs 36 %; *p* = 0.00) and partners (50 % vs 24 %; *p* = 0.00) avoid intercourse significantly more frequently in cases with POP compared with SUI. This did not have a significant impact on quality of life.

**Conclusions:**

The impact of bothersome SUI or POP on sexual function was found to be similar, but patient and partner avoidance in women with POP was greater than those with SUI.

## Introduction

Pelvic organ prolapse (POP) and stress urinary incontinence (SUI) are disorders of the pelvic floor that affect about a third of community dwelling women with a significant impact on a woman’s quality of life [1, 2]. Sexual problems have been well known in women attending a urogynaecology clinic, ranging from 37 to 64 % [3, 4]. Sexual problems commonly described in women with POP or SUI include disorders of desire, arousal, orgasm and dyspareunia. Disorders in the male partner are also seen, including premature ejaculation and erectile dysfunction [5]. A community-based survey found no difference between sexual activity and satisfaction compared with women without pelvic floor dysfunction [6].

There is conflicting evidence in the literature regarding the effect of POP and SUI on sexual function. Patients with prolapse may have coexistent urinary symptoms and vice versa owing to the close anatomical relationship and also the similar underlying pathophysiology. Most studies therefore report on women with mixed symptoms of both POP and SUI. Some studies report no difference in the sexual function in women with prolapse or incontinence [7]; others report that prolapse is more likely to affect sexual relations than incontinence [8, 9], whereas some studies found that women with urinary incontinence were more likely to report low libido, vaginal dryness and dyspareunia [10]. All these studies have been in patients with both prolapse and urinary symptoms, making it difficult to differentiate if there are any actual differences in sexual function between these two subgroups. Also, most studies failed to use validated questionnaires, casting doubt on the validity of findings.

The aim of this study was to evaluate female sexual function in two subsets of patients bothered by POP and SUI, without an overlap of symptoms, to reduce the confounding bias due to these factors.

## Materials and methods

This was a retrospective cohort questionnaire based study conducted at a tertiary hospital in the UK. All patients attending the urogynaecology department complete an electronic pelvic floor assessment questionnaire as part of routine clinical care before being reviewed by a clinician. The electronic pelvic floor assessment questionnaire (ePAQ), a validated symptom-specific and quality-of-life questionnaire was used for this purpose [11]. Pre-operative data on sexual function were collected on all sexually active women who underwent surgery for SUI or POP, in the period from January 2008 to December 2012. A post hoc power analysis was performed and the available sample size had a greater than 80 % power of detecting 3-point differences at the 5 % (two-sided) level with a standard deviation of 5 points. This instrument provides symptom assessment in four dimensions: urinary, bowel, vaginal and sexual. Patients who had concurrent prolapse and urinary incontinence were excluded from the study. Women scheduled for surgery were selected rather than those opting for conservative treatment (physiotherapy or ring pessary) as it identified a cohort of women who were more bothered or were likely to have a greater severity of prolapse or incontinence. All ePAQ data were collected preoperatively. Women undergoing SUI surgery had no symptoms of POP on clinical examination. This was further confirmed by the ePAQ scores, which showed a negative screen for the prolapse item in these patients. They all had urodynamically proven SUI without any detrusor overactivity. Patients with POP had a stage 2 prolapse or greater (Ba or C at or beyond 0 on POP-Q), without any bothersome urinary symptoms, including urinary incontinence. These patients were screened as being negative for the impact of stress urinary incontinence on urinary domain related quality of life item on the ePAQ. It is not routine practice in our unit to perform a stress reduction test; thus, data on occult stress incontinence were not available.

The sexual dimension of the ePAQ has five domains, which include urinary, bowel, vaginal, dyspareunia and general sex life. Within both groups, both item and bother scores from the ePAQ were collected for the urinary and vaginal domains for analysis. These scored on an ordinal scale from 0 to 3, where 0 represents “Never” and 3 represents “All the time”. SUI and POP have been shown to have an impact on sexual function in different ways [12]. In the SUI group, scores were collected for items including overall impact due to incontinence, penetration and orgasm incontinence, and post-coital urinary tract infections (UTI). In the POP group, the items dryness of the vagina, discomfort and pain, lack of sensation, tightness and obstruction were measured. A comparison of the two subgroups in terms of overall impact, avoidance of intercourse by patient and partner and patient anxiety were done. All analyses were performed using SPSS version 19 and non-parametric data were compared using the Mann–Whitney* U* test and demographic data were compared using Student’s *t* test.

This project was registered as a service evaluation project with Sheffield Teaching Hospitals NHS Foundation Trust. Data used for this study were taken only from patients who answered “Yes” to the final two items of the questionnaire:

- “(D2a) Are you willing to allow confidential use of your answers in order to evaluate the care you receive?
- (D2b) Are you willing to allow confidential use of your answers in order to check how this questionnaire is working?”

## Results

One hundred and eighty-four women who underwent a mid-urethral sling (TVT) procedure and were sexually active, completed the ePAQ pre-operatively. The age range was between 33 and 77 years, with a median age of 49. One hundred and fifty-nine women in the prolapse group who underwent pelvic floor repair, which included anterior repair and/or posterior repair, with or without a hysterectomy, were sexually active and completed the ePAQ. The age range of these women was 27 to 78 years, with a median age of 59. Although the median age of patients with prolapse appeared to be higher than that of patients with stress urinary incontinence, this was not found to be statistically significant (*p* = 0.869).

On comparison of the overall incidence of interference of sexual activity due to POP and SUI symptoms, the incidence was found to be higher in the POP group (*n* = 113;7 1 %) compared with the SUI group (*n* = 98; 53 %). However, the mean ePAQ scores were similar for POP (1.56 ± 1.05) and SUI (1.62 ± 1.07) and did not reach any statistical significance (*p* = 0.703). On comparing the avoidance of sexual activity, 116 patients with POP avoided sexual activity (73 %) compared with 66 women with SUI (36 %; *p* = 0.000). However, the mean impact scores for avoidance of sexual activity were similar in these two groups (*p* = 0.609). With regard to avoidance of sexual activity by the partner due to patient’s symptoms, the incidence was higher in the POP group (*n* = 80; 50 %) compared with the SUI group (*n* = 44; 24 %). Again, this did not reach statistical significance with regard to impact on quality of life (*p* = 0.820). Patient anxiety was found to be similar in the POP (*n* = 121; 76 %) and SUI (*n* = 116; 63 %) groups respectively. These results are shown in Table 1.

**Table 1:** Impact of urinary incontinence and pelvic organ prolapse (*POP*) on sexual function

Item	POP (*n* = 159) incidence (%)	SUI (*n* = 184) incidence (%)	POP ePAQ scores, mean (± SD)	SUI ePAQ scores, mean (± SD)	*p* value ePAQ scores
Incidence and overall impact on sexual activity	71	53	1.56 (1.05)	1.62 (1.07)	0.703
Patient avoids intercourse	73	36	1.34 (1.09)	0.90 (0.92)	0.000
Impact of patient avoidance	67	51	1.55 (1.07)	1.61 (1.00)	0.609
Partner avoids intercourse	50	24	0.85 (1.01)	0.42 (0.76)	0.000
Impact of partner avoidance	45	24	1.11 (1.11)	1.18 (1.20)	0.820
Patient anxiety	76	63	1.43 (1.11)	1.33 (1.13)	0.4114

Mann–Whitney* U* test used for calculation of *p* values

*SUI* stress urinary incontinence,* ePAQ* electronic pelvic floor assessment questionnaire

In the SUI group, 40 % (74 out of 184) complained of orgasm incontinence and 31 % (57 out of 184) complained of penetration incontinence. Thirty per cent of women (55 out of 184) reported post-coital UTIs. With regard to the impact of specific items, the impact of urinary leakage during sex (1.62 ± 1.07) was the highest, followed by impact of post-coital UTI (1.42 ± 0.94). In the POP group, 53 % (84/ out of 59) complained of dryness in the vagina, 60 % (95 out of 159) complained of lack of sensation, 56 % (89 out of 159) had vaginal discomfort, 18 % (29 out of 159) complained of tightness and 59 % (94 out of 159) complained of obstruction. Of the specific vagina items, the highest impact score (1.36 ± 1.18) was for the feeling of vaginal obstruction due to POP and the tightness of the vagina caused the least impact on sexual activity (0.42 ± 0.84). Specific symptoms in the subgroup affecting sexual function are shown in Table 2.

**Table 2:** Incidence of bladder-specific and prolapse-specific symptoms that influence sexual function

Subgroup	Symptom	Incidence (%)	Mean impact score
SUI (*n* = 184)	Orgasm incontinence	40	0.79 ± 1.18
	Penetration incontinence	31	0.66 ± 1.16
	Post-coital urinary tract infections	30	1.42 ± 0.94
POP (*n* = 159)	Dryness in the vagina	53	1.05 ± 0.97
	Lack of sensation	60	1.21 ± 1.03
	Discomfort	56	1.19 ± 1.04
	Tightness	18	0.42 ± 0.84
	Obstruction	59	1.36 ± 1.18

## Discussion

Pelvic floor symptoms have a similar impact on sexual function in women with bothersome POP and SUI. Patient and partner avoidance of intercourse was greater in women with POP compared with women with SUI, but this did not have an impact on quality of life.

This is the largest study looking at specific pelvic floor problems and their comparative impact on sexual function. The study by Barber et al. [8] included only 32 patients with prolapse and 29 patients reporting lower urinary tract symptoms including urinary incontinence. Similarly, the study by Weber et al. [7] only reported on 80 women. They reported that when prolapse interfered with sexual intercourse, women had significantly more advanced prolapse and more incontinence episodes than when POP did not interfere with intercourse. Other studies with larger sample sizes [6, 9, 10, 13, 14] had mixed populations, looking at women with both pelvic organ prolapse and urinary incontinence to varying degrees, and sometimes those not needing any treatment. It is difficult, therefore, to extrapolate from these studies the exact effect of these individual symptoms on sexual function or indeed compare the two. Another strength of our study is that all the patients included had significant symptoms, as they were on a waiting list for surgery and were therefore significantly bothered by their pelvic floor symptoms.

A study by Ozel et al. [15] looked at patients who suffered SUI with and without POP. The study found that women with POP in conjunction with SUI are more likely to report decreased libido, decreased sexual excitement and difficulty achieving orgasm during intercourse than women with UI alone. The sample size was significantly less than in our study and this study presupposes that SUI causes a baseline level of sexual dysfunction.

Our data were similar to those reported by Ellerkmann et al. [16] and Barber et al. [8], who found that women with bothersome POP avoided intercourse. This could be because POP causes obstructive symptoms during intercourse, which is apparent both to the patient and the partner, hence why they are more reluctant to engage in sexual activity. In addition, the presence of prolapse may affect a woman’s self-image, resulting in a reluctance to be intimate with her partner. For partners the avoidance may be related to a fear of causing worsening of the prolapse and their partner’s symptoms.

The main weakness of our study is that this was a retrospective study; thus, it is difficult to comment on other factors that may have an impact on sexual function, such as the number of women who were menopausal, the proportion of women on HRT or local oestrogen replacement therapy. In addition, we did not have any data on the partner and their sexual status with regard to inherent problems that may have an impact on sexual function. In the POP group we excluded women with stress urinary incontinence, we did not exclude women with micturition or urgency symptoms, as these data were not available. This may be considered a shortcoming; however, we feel that these symptoms were more likely to be related to the prolapse. There is also the absence of a control group to compare the baseline incidence of sexual dysfunction in the general population compared with our study group. It could be argued that the sexual problems experienced by patients in the study group may not be linked exclusively to their pelvic floor problems.

The results of our study enhance the understanding of sexual dysfunction in women with pelvic floor dysfunction. It also dispels several myths related to these conditions, including the fact that POP per se results in a greater impact of various aspects of sexual function compared with SUI. This does not appear to be the case. Our study allows better counselling of women deciding to proceed with surgery for these conditions and allows us to give them more realistic expectations of what symptoms are related to individual pelvic floor problems. The role of conservative treatments such as pelvic floor muscle training cannot be underestimated [17]. We have previously reported on the impact of the surgical treatment of POP [18, 19] and SUI [20, 21] on sexual function and the results are very reassuring and similar to those reported by other authors [22]. This study also emphasises the need for healthcare professionals to assess sexual health in women with bothersome POP and SUI. It has been demonstrated that there is definite scope for improvement in this regard [23]. Irrespective of age, patients are bothered to varying degrees by the impact of these conditions on their sexual function.

Sexual dysfunction is very complex and the impact of pelvic floor problems is unlikely to be a linear effect. The history of progression of sexual dysfunction in these women needs to be further explored, taking into account all the variables that might affect sexual function. Larger well-designed studies looking at demographic and childbirth data in addition to hormone status are required to understand the pathophysiology of sexual dysfunction in women with pelvic floor disorders and to identify preventative and therapeutic strategies.
